# Supplementary material for: Hot spot prediction in protein-protein interactions by an ensemble system
Source: BMC Syst Biol. 2018 Dec 31;12(Suppl 9):132. doi: 10.1186/s12918-018-0665-8 (PMC6311905; doi:10.1186/s12918-018-0665-8)
Supplement: Supplementary file 2 — All relevant MATLAB code. A simple Matlab implement of our predictor. (ZIP 19,742 kb) [file 12918_2018_665_MOESM2_ESM.zip › Additional file 2/Read ASA/ASA website/dbMPIKT/1BJ1V.html]

 NetSurfP - Protein Surface Accessibility and Secondary Structure Predictions

  

|  |  |  |
| --- | --- | --- |
|  |  | NetSurfP - Protein Surface Accessibility and Secondary Structure Predictions  Technical University of Denmark |

  


---

```
# For publication of results, please cite:
# A generic method for assignment of reliability scores applied to solvent accessibility predictions.
# Bent Petersen, Thomas Nordahl Petersen, Pernille Andersen, Morten Nielsen and Claus Lundegaard
# BMC Structural Biology 2009, 9:51 doi:10.1186/1472-6807-9-51
#
# Column 1: Class assignment - B for buried or E for Exposed - Threshold: 25% exposure, but not based on RSA
# Column 2: Amino acid
# Column 3: Sequence name
# Column 4: Amino acid number
# Column 5: Relative Surface Accessibility - RSA
# Column 6: Absolute Surface Accessibility
# Column 7: Z-fit score for RSA prediction
# Column 8: Probability for Alpha-Helix
# Column 9: Probability for Beta-strand
# Column 10: Probability for Coil
E G  1BJ1V                  1    0.927  72.963  -0.774   0.003   0.003   0.994
E Q  1BJ1V                  2    0.676 120.662  -1.159   0.058   0.017   0.925
E N  1BJ1V                  3    0.558  81.618  -0.912   0.184   0.043   0.773
E H  1BJ1V                  4    0.467  84.856   0.013   0.354   0.048   0.598
E H  1BJ1V                  5    0.403  73.306  -0.657   0.428   0.171   0.402
E E  1BJ1V                  6    0.492  85.883   0.533   0.453   0.248   0.299
B V  1BJ1V                  7    0.194  29.895   0.469   0.478   0.309   0.214
B V  1BJ1V                  8    0.170  26.068   1.236   0.453   0.248   0.299
E K  1BJ1V                  9    0.461  94.910   1.511   0.538   0.173   0.289
B F  1BJ1V                 10    0.119  23.783  -0.015   0.701   0.107   0.192
B M  1BJ1V                 11    0.110  21.951   0.212   0.779   0.100   0.120
E D  1BJ1V                 12    0.429  61.776   0.312   0.779   0.100   0.120
B V  1BJ1V                 13    0.194  29.772  -0.162   0.779   0.100   0.120
B Y  1BJ1V                 14    0.198  42.270   0.291   0.561   0.047   0.393
E Q  1BJ1V                 15    0.520  92.783   0.522   0.354   0.048   0.598
E R  1BJ1V                 16    0.587 134.400   0.386   0.113   0.043   0.844
B S  1BJ1V                 17    0.135  15.834  -2.609   0.052   0.084   0.864
E Y  1BJ1V                 18    0.466  99.627   0.593   0.021   0.279   0.699
B C  1BJ1V                 19    0.047   6.613  -1.786   0.021   0.451   0.528
E H  1BJ1V                 20    0.423  76.907   1.183   0.004   0.514   0.481
E P  1BJ1V                 21    0.351  49.821  -0.437   0.005   0.336   0.660
E I  1BJ1V                 22    0.355  65.749   0.920   0.004   0.420   0.576
E E  1BJ1V                 23    0.641 111.965  -0.469   0.004   0.514   0.481
E T  1BJ1V                 24    0.293  40.681   0.750   0.002   0.816   0.182
B L  1BJ1V                 25    0.220  40.355   0.496   0.001   0.959   0.040
B V  1BJ1V                 26    0.068  10.513   1.176   0.001   0.959   0.040
E D  1BJ1V                 27    0.387  55.810   0.720   0.001   0.959   0.040
B I  1BJ1V                 28    0.040   7.382  -0.043   0.018   0.846   0.136
E F  1BJ1V                 29    0.276  55.393   0.484   0.003   0.718   0.279
E Q  1BJ1V                 30    0.473  84.460  -0.470   0.004   0.514   0.481
E E  1BJ1V                 31    0.535  93.517  -0.577   0.022   0.359   0.619
E Y  1BJ1V                 32    0.285  60.862  -0.204   0.004   0.085   0.910
E P  1BJ1V                 33    0.455  64.607  -1.385   0.005   0.045   0.951
E D  1BJ1V                 34    0.635  91.475  -0.862   0.018   0.047   0.935
E E  1BJ1V                 35    0.519  90.582  -0.785   0.018   0.047   0.935
B I  1BJ1V                 36    0.265  49.006  -0.889   0.018   0.088   0.893
E E  1BJ1V                 37    0.315  55.100  -0.692   0.021   0.279   0.699
B Y  1BJ1V                 38    0.162  34.705  -0.085   0.004   0.514   0.481
B I  1BJ1V                 39    0.176  32.505   0.203   0.002   0.816   0.182
B F  1BJ1V                 40    0.065  12.985  -0.172   0.002   0.816   0.182
B K  1BJ1V                 41    0.239  49.080   0.769   0.004   0.514   0.481
B P  1BJ1V                 42    0.062   8.755  -0.335   0.004   0.197   0.799
E S  1BJ1V                 43    0.278  32.593  -0.414   0.004   0.197   0.799
B C  1BJ1V                 44    0.058   8.185  -0.354   0.004   0.616   0.381
B V  1BJ1V                 45    0.094  14.371   0.520   0.011   0.918   0.071
E P  1BJ1V                 46    0.265  37.533   0.687   0.011   0.918   0.071
B L  1BJ1V                 47    0.050   9.082   0.704   0.018   0.846   0.136
E M  1BJ1V                 48    0.270  53.987   0.410   0.021   0.756   0.223
B R  1BJ1V                 49    0.248  56.907  -0.224   0.023   0.655   0.322
B C  1BJ1V                 50    0.029   4.100  -0.764   0.021   0.451   0.528
B G  1BJ1V                 51    0.124   9.782  -1.485   0.022   0.359   0.619
B G  1BJ1V                 52    0.094   7.421  -3.482   0.005   0.262   0.733
B C  1BJ1V                 53    0.070   9.800  -0.507   0.021   0.279   0.699
B C  1BJ1V                 54    0.040   5.686  -0.008   0.020   0.205   0.775
B N  1BJ1V                 55    0.313  45.809  -0.392   0.018   0.047   0.935
E D  1BJ1V                 56    0.482  69.514  -1.646   0.018   0.019   0.964
E E  1BJ1V                 57    0.655 114.411  -0.666   0.018   0.019   0.964
E G  1BJ1V                 58    0.483  38.044  -0.413   0.004   0.085   0.910
B L  1BJ1V                 59    0.217  39.714   0.711   0.004   0.420   0.576
E E  1BJ1V                 60    0.442  77.165   0.874   0.002   0.816   0.182
B C  1BJ1V                 61    0.051   7.090  -1.553   0.001   0.900   0.099
E V  1BJ1V                 62    0.335  51.520   0.991   0.002   0.816   0.182
B P  1BJ1V                 63    0.221  31.360  -1.194   0.004   0.616   0.381
E T  1BJ1V                 64    0.372  51.583   0.017   0.004   0.420   0.576
E E  1BJ1V                 65    0.514  89.866  -0.168   0.004   0.616   0.381
E E  1BJ1V                 66    0.493  86.197  -0.212   0.002   0.816   0.182
E S  1BJ1V                 67    0.352  41.243   0.202   0.002   0.816   0.182
E N  1BJ1V                 68    0.516  75.572   0.073   0.002   0.816   0.182
B I  1BJ1V                 69    0.162  29.952   1.184   0.001   0.900   0.099
E T  1BJ1V                 70    0.420  58.254   0.252   0.001   0.959   0.040
B M  1BJ1V                 71    0.115  23.092   1.092   0.001   0.959   0.040
E Q  1BJ1V                 72    0.299  53.437   1.190   0.001   0.959   0.040
B I  1BJ1V                 73    0.045   8.251  -0.047   0.001   0.959   0.040
B M  1BJ1V                 74    0.216  43.222  -0.045   0.001   0.959   0.040
B R  1BJ1V                 75    0.204  46.762  -0.451   0.001   0.959   0.040
B I  1BJ1V                 76    0.103  19.073  -0.046   0.002   0.816   0.182
E K  1BJ1V                 77    0.433  89.089   0.386   0.004   0.514   0.481
E P  1BJ1V                 78    0.440  62.379  -0.571   0.004   0.197   0.799
E H  1BJ1V                 79    0.561 102.082  -1.429   0.005   0.045   0.951
E Q  1BJ1V                 80    0.500  89.282  -1.241   0.005   0.045   0.951
E G  1BJ1V                 81    0.543  42.718  -0.847   0.004   0.085   0.910
E Q  1BJ1V                 82    0.462  82.513  -1.060   0.004   0.138   0.858
E H  1BJ1V                 83    0.582 105.884  -0.287   0.004   0.420   0.576
E I  1BJ1V                 84    0.413  76.386   0.175   0.002   0.816   0.182
B G  1BJ1V                 85    0.189  14.898   0.540   0.001   0.959   0.040
E E  1BJ1V                 86    0.506  88.433   1.026   0.001   0.959   0.040
B M  1BJ1V                 87    0.054  10.805   1.105   0.001   0.900   0.099
E S  1BJ1V                 88    0.328  38.406   1.550   0.001   0.900   0.099
B F  1BJ1V                 89    0.080  16.136   0.306   0.002   0.816   0.182
E L  1BJ1V                 90    0.482  88.254   0.649   0.003   0.718   0.279
E Q  1BJ1V                 91    0.501  89.550   0.220   0.004   0.514   0.481
B H  1BJ1V                 92    0.235  42.728   0.093   0.021   0.279   0.699
E N  1BJ1V                 93    0.511  74.854   0.352   0.005   0.336   0.660
E K  1BJ1V                 94    0.639 131.483   1.756   0.004   0.616   0.381
B C  1BJ1V                 95    0.069   9.702  -0.256   0.003   0.718   0.279
E E  1BJ1V                 96    0.520  90.774   1.152   0.001   0.900   0.099
B C  1BJ1V                 97    0.076  10.684  -1.196   0.002   0.816   0.182
E R  1BJ1V                 98    0.441 101.035   1.516   0.003   0.718   0.279
E P  1BJ1V                 99    0.396  56.221  -1.258   0.004   0.616   0.381
E K  1BJ1V                100    0.456  93.861   0.896   0.005   0.262   0.733
E K  1BJ1V                101    0.645 132.656  -0.789   0.018   0.047   0.935
E D  1BJ1V                102    0.957 137.889   0.194   0.000   0.000   0.999

Explain the output.  Go back.
```

---
